# Gold nanocolloid–protein interactions and their impact on β-sheet amyloid fibril formation†

**DOI:** 10.1039/c7ra11219j

**Published:** 2018-01-03

**Authors:** Heloise R. Barros, Maria Kokkinopoulou, Izabel C. Riegel-Vidotti, Katharina Landfester, Héloïse Thérien-Aubin

**Affiliations:** Max Planck Institute for Polymer Research 55128 Mainz Germany therien@mpip-mainz.mpg.de; Departamento de Química, Universidade Federal do Paraná CxP 19081, CEP 81531-980 Curitiba PR Brazil

## Abstract

The influence of the presence of small molecules and nanoparticles on the mechanism of amyloid fibril formation has attracted attention because amyloid protein fibrils are associated with degenerative diseases. Here, we studied the interaction between gold nanoparticles (AuNPs) and a model protein (lysozyme). Both the formation of amyloid fibrils in the presence of gold nanoparticles, as well as the interaction between lysozyme and the amyloid fibrils with AuNPs, were investigated to gain an understanding of the distinct behaviour of lysozyme in its fibrillar and globular form. It was observed that the presence of AuNPs delayed the unfolding of α-helixes present in the globular lysozyme and the formation of the amyloid fibrils. However, the addition of AuNPs was also associated with a larger amount of β-sheet structures in the system once equilibrium was reached. Furthermore, the results showed that the driving force of the interaction between AuNPs and lysozyme in its fibrillar and globular forms was significantly different, and that the interaction of AuNPs with the preformed lysozyme amyloid fibrils led to a structural change in the protein.

## Abstract 2

Formation of amyloid protein fibrils is associated with degenerative diseases. Here, the interaction mechanism between globular and fibrillar proteins with AuNPs were investigated in order to potentially control and reverse the fibrillation process.

## Graphical abstract

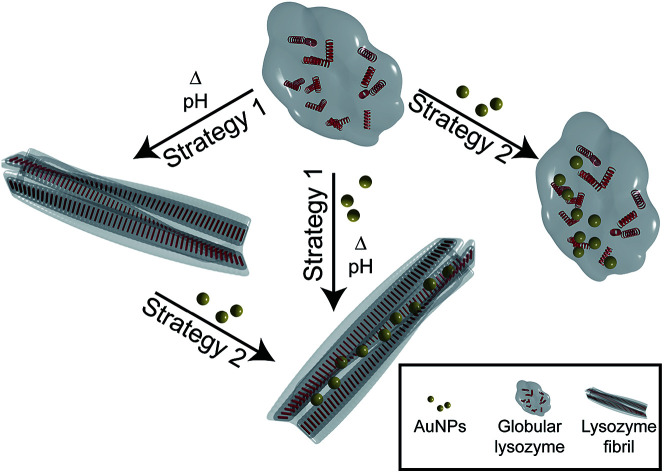

## Introduction

Amyloid fibrils are formed by the self-assembly of protein aggregates composed of highly ordered β-sheet structures and are associated with degenerative diseases such as Alzheimer's, Parkinson's, and many others.^1–3^ These aggregates are formed by misfolded proteins with exposed hydrophobic groups.^3^ One of the key challenges associated with the formation of amyloid fibrils is not only to prevent or disrupt the aggregation of the proteins but also to reverse the misfolding process and to return the proteins to their original conformation. However, to achieve control over the fibrillation process, we are still in need of a deeper understanding of the different mechanisms associated with the fibrillation process.

The mechanism of amyloid fibril formation is complex even for samples involving only the protein.^4–7^*In vitro*, amyloid fibrils could be rapidly produced under mildly denaturing conditions.^8^ Nucleation could occur in a homogeneous manner or *via* a secondary nucleation mechanism where fibers act as nucleation points. Furthermore, during the fibrillation process, the addition of protein is in competition with the fragmentation of the existing fibers. Moreover, the presence of additive from small molecules (vitamins,^9^ polyphenols,^10^ flavonoids,^11^ metal chelators,^12^…) to larger species such as polypeptides^2^ and other molecular chaperons^13^ can interfere with the fibrillation process by inhibiting one or many of the multiple steps involved in the formation of β-amyloid fibers.

The addition of nanoparticles to the protein solution before fibrillation complexifies the mechanism further.^14,15^ Upon the addition of nanoparticles to any protein solution a rapid adsorption of proteins occurs at the interface between the nanoparticle and its environment leading to the formation of a protein corona around the nanoparticle.^16–19^ The adsorption of proteins on the surface of the nanoparticle has a dual effect, as it affects the behaviour and fate of the particles^20–22^ but also contributes to the unfolding and clustering of the proteins.^14,23^ These conformational changes of the protein have the potential to affect the mechanism of fibril formation. This is why the effect on protein fibrillation of a variety of nanoparticles, such as polymers,^14,24^ proteins,^25^ magnetic iron oxide,^15,26,27^ or noble metal^1,27–33^ has been examined. The interaction at the interface of a nanoparticle and the proteins brings even more complexities to the fibrillation mechanism; for example, the surface of gold nanoparticles has been shown to interact favourably with the amino groups of the protein.^34,35^ Additionally, the formation of amyloid fibrils is associated with a conformational change of the protein exposing more amino, amide and carboxyl groups, that were previously buried in the protein core. Thus, the formation of β-amyloid fibers increases the number of potential protein–AuNPs binding sites.^3^ The size, shape, surface charge, hydrophilicity, and concentration of AuNPs are determinant factors in the mechanism of amyloid fibrils formation.^14,30,35^ Consequently, the effect of AuNPs on the amyloid fibrils is complex and ambiguous. And until now, experimental results have shown diverging trends. For example, the addition of AuNPs could either increase^1^ or decrease^32^ the rate of fibrils formation and the overall effect of the nanoparticles on the fibrillation process. To fully understand the effect of nanoparticles on the formation and stability of β-amyloid fibrils, not only their effect on the fibrillation process need to be better understood but also the interactions between the fibrils themselves and those nanocolloids.

**Fig. 1:**
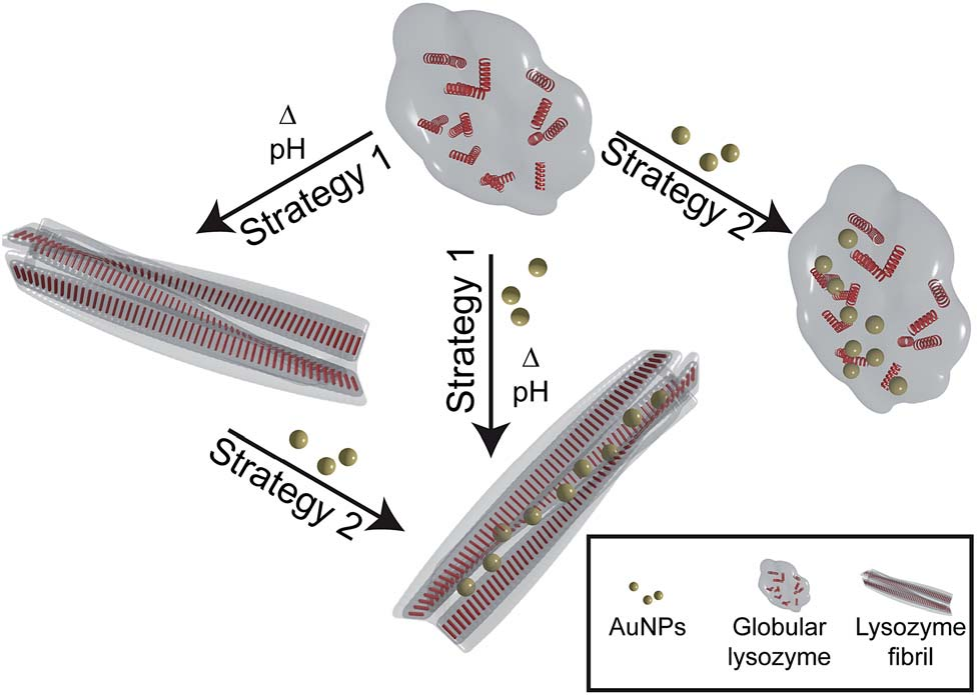
Schematic illustration of the two different strategies used to study the interaction between AuNPs and lysozyme. Strategy 1: lysozyme fibrillation in presence of AuNPs. Strategy 2: interaction study between AuNPs and lysozyme globules and fibrils. Not drawn to scale.

Here, we investigate the interaction and the effect of AuNPs on the formation and structure of amyloid lysozyme fibrils. Lysozyme was used for this purpose because of its involvement in amyloidosis^36^ and its wide use as an *in vitro* model for amyloid fibril formation.^37,38^ To investigate the difference between the interaction of globular and fibrillar lysozyme with gold nanoparticles, two different strategies were implemented (Fig. 1). In the first case, the fibrillation of lysozyme was carried out in presence of AuNPs to investigate their interactions with globular lysozyme and the influence of the addition of AuNPs on the mechanism and kinetics of fibrils formation (strategy 1). Alternatively, AuNPs were added to preformed β-amyloid lysozyme fibrils to study the interaction involved between AuNPs and fibrils (strategy 2). Those two strategies together would provide the necessary information to clarify the difference in the interactions of AuNPs with both native lysozyme and β-amyloid lysozyme fibrils.

## Results and discussion

### Lysozyme fibrillation in presence of AuNPs

**Fig. 2:**
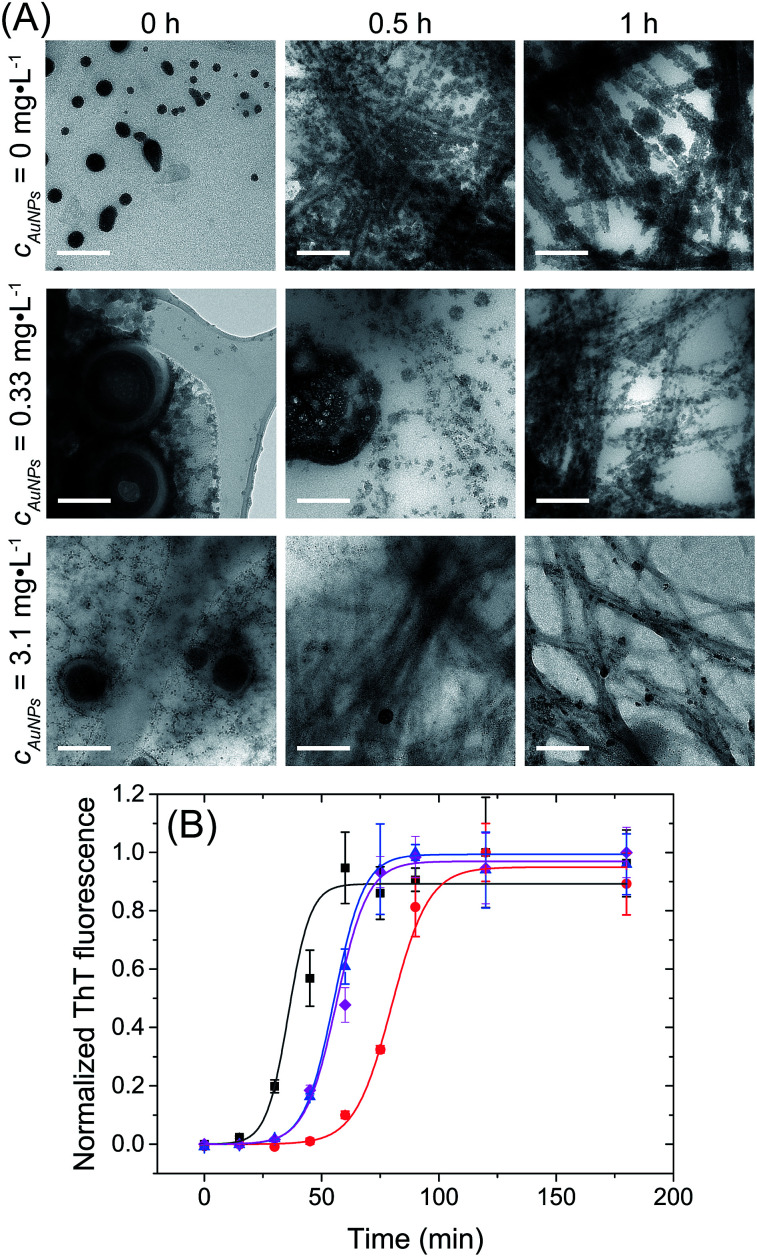
Lysozyme fibrillation in presence of AuNPs. (A) TEM micrographs of ionic liquid embedded samples, prepared during the lysozyme fibrillation at *t*_SA_ = 0, 0.5 and 1 h with *c*_AuNP_ = 0 mg L^−1^, *c*_AuNP_ = 0.33 mg L^−1^ and *c*_AuNP_ = 3.1 mg L^−1^. Scale bar: 200 nm. (B) Kinetic profile of fibrils formation followed by ThT fluorescence with *c*_AuNP_ = 0 mg L^−1^ (black), *c*_AuNP_ = 0.07 mg L^−1^ (red), *c*_AuNP_ = 0.33 mg L^−1^ (blue) and *c*_AuNP_ = 3.1 mg L^−1^ (pink). *λ*_ex_ = 440 nm. The lines are fits to eqn (1).

Fig. 2 shows the evolution of the structures observed in lysozyme/AuNPs suspensions over the course of the lysozyme fibrillation in presence of a range of AuNPs concentration (*c*_AuNP_) varying from 0 to 3.1 mg L^−1^ (strategy 1). In every case, a different concentration of gold was added to a solution of globular lysozyme, then the fibrillation was triggered by the addition of HCl (pH = 2) followed by incubation at 60 °C. In these conditions, the fibrillation of the lysozyme is observed in absence of protein hydrolysis as evidenced by electrophoresis (ESI, Fig. S5†). During this process, amyloid fibrils (aggregates composed of β-sheet structures) were formed and the fibrillation was accompanied by an increase in the scattering of the solution. The kinetics of the lysozyme fibrillation was monitored by transmission electron microscopy (TEM) images (Fig. 2A) at different time of self-assembly (*t*_SA_) and by Thioflavin T (ThT) fluorescence (Fig. 2B). ThT is a dye molecule that specifically binds to β-sheet structures.^1,2^ The TEM images show that at *t*_SA_ = 0 h, only globular protein aggregates were observed in all samples. As *t*_SA_ increases, the formation of protofibrils and fibrils was observed for every sample. At *t*_SA_ = 0.5 h, the presence of protofibrils was more pronounced for the fibrillation of lysozyme at *c*_AuNP_ = 0 mg L^−1^ where all the globular protein aggregates had been consumed, while samples at *c*_AuNP_ = 0.33 mg L^−1^ and 3.1 mg L^−1^ displayed mainly globular protein aggregates coexisting with some fibrils. At this stage, no free AuNPs were observed, all the AuNPs were embedded in either lysozyme globules or fibrils. Finally, at *t*_SA_ = 1 h, in absence of AuNPs only lysozyme fibrils were observed, however in presence of AuNPs, some lysozyme globules were detected. TEM tomography was used to precisely locate the AuNPs. The 3D reconstructions (ESI, Fig. S2†) demonstrate that the AuNPs were distributed through the entire volume of reconstructed sites; an evidence that the AuNPs are inside both the globular and fibrillar structures.

To better understand the effect of AuNP of the formation of lysozyme amyloid fibers, the kinetics of the protein reorganization was followed by ThT fluorescence at different *c*_AuNPs_ (0, 0.07, 0.33, and 3.1 mg L^−1^). The fibrillation kinetics, as seen by the ThT fluorescence, displayed a sigmoidal profile, typical of the formation of amyloid fibrils.^39^ The sigmoidal shape of ThT fluorescence curve consists of an initial lag phase, during which the unfolding of the α-helix present in the lysozyme globules and nucleation of the fibrils occurs. This is followed by a growth phase associated with the protein–protein interactions leading to the growth of the amyloid fibrils, and finally, a stable plateau phase where the fibrillation process is completed and the highest amount of β-sheet is achieved. The fibrillation kinetics was described by eqn (1):^4^1
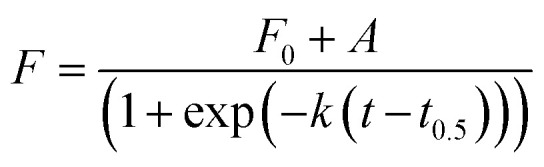
where *F*_0_ is the fluorescence intensity, *A* is the amplitude of the transition, *k* is the apparent rate of fibril formation, *t* is the time of self-assembly (or incubation), and *t*_0.5_ is the midpoint of the growth phase. The lag time was defined as *t*_lag_ = *t*_0.5_ − 1/2*k*. Those parameters are dependent on the conditions used for the fibrillation as protein concentration, solvents, temperature and pH.^38,40,41^Eqn (1) fitted all curves adequately, providing the values of *t*_lag_ and apparent rate of fibril formation (*k*) summarised in Table S1 (ESI†).

At *t*_SA_ = 0 h, no fluorescence was observed in any sample, indicating that no β-sheet aggregates or fibrils were formed at this stage. The lag phase, the stage prior to fibril formation defined by the lag time (*t*_lag_), was shown to depend on the concentration of AuNPs. The addition of AuNPs led to an increase of *t*_lag_ from 39 ± 1 min (*c*_AuNP_ = 0 mg L^−1^) to 79 ± 4 min (*c*_AuNP_ = 0.07 mg L^−1^). The TEM images obtained at *t*_SA_ = 0.5 h and 1 h correspond to the elongation phase of fibrils. This stage was delayed in presence of AuNPs at 0.33 and 3.1 mg L^−1^, where the coexistence of globular and fibrils protein aggregates could be observed.

Longer *t*_lag_ values were obtained in presence of AuNPs in all concentrations. The longer *t*_lag_ fibrillation is correlated to the formation of large globular lysozyme aggregates in the presence of AuNPs, as observed in TEM images at *t*_SA_ = 0 h. This suggests that the interaction between AuNPs and globular lysozyme lead to the formation of large domains where the lysozyme was partially adsorbed onto the surface of the AuNP. This led to a decrease of the effective concentration of free lysozyme. The decrease in the concentration of lysozyme in solution is known to increase the lag-time of the fibrillation process.^4,42^ This depletion of the free protein available to undergo nucleation has been previously observed for other types of nanoparticles.^24^

While an increase in the lag-time is observed for every sample containing AuNPs, the effect of *c*_AuNP_ was not linear. The nanoparticles themselves could act as nucleation point.^14^ This phenomenon lead to a turnover point where the lag-time of the fibrillation increases.^24^ While the decrease in the effective concentration of free lysozyme decrease when the concentration of AuNP increases leading to a reduction in the rate of primary homogeneous nucleation, the rate a secondary nucleation by the AuNP surface increases, the clusters of protein adsorbed on the surface of the nanoparticles could act as a seed for the fibrillation process.^24^

**Fig. 3:**
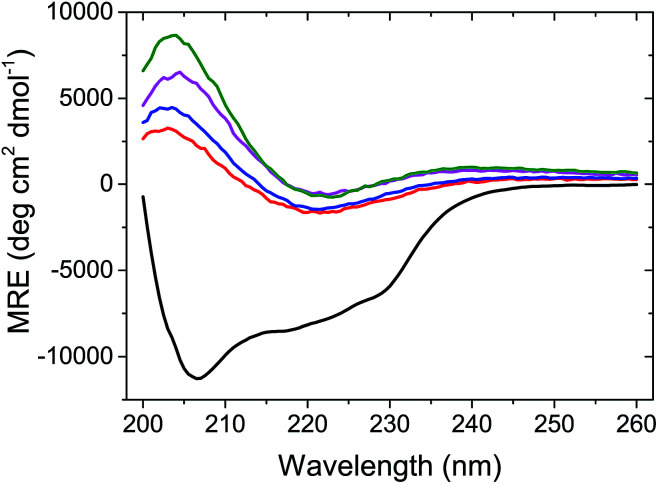
CD spectra of lysozyme globular (black) and fibrils *c*_AuNP_ = 0 mg L^−1^ (red), *c*_AuNP_ = 0.07 mg L^−1^ (blue), *c*_AuNP_ = 0.33 mg L^−1^ (pink) and *c*_AuNP_ = 3.1 mg L^−1^ (green). The spectra were obtained at the end of fibrillation.

At the end of the fibrillation process, after *t*_SA_ = 5 h, circular dichroism (CD) measurements were performed to analyse the changes in the protein conformation after fibrillation (Fig. 3). The spectrum of globular lysozyme shows negative peaks at 208 and 222 nm typical of the α-helix conformation.^43^ After the fibrillation, the spectra of all samples displayed the spectral signature typical of β-sheet characterised by a positive signal at 205 nm and a broad negative signal at *ca.* 225 nm.^43^ This is in accordance with the proposed mechanism of β-amyloid fibril formation^9^ where the lysozyme in the α-helix-rich globules first unfolds, followed by the formation of fibrils containing protein in a β-sheet conformation.

Furthermore, Fig. 3 shows that molar residue ellipticity (MRE) of the lysozyme solution increased with the addition of a larger amount of AuNPs. This, in conjunction with the decrease in *t*_lag_ suggests that the AuNPs promote the formation of amyloid fibrils.

### Interactions between AuNPs and lysozyme fibrils

The second type of AuNP/lysozyme interaction investigated was between the preformed fibrils and the AuNPs (Fig. 1). Solutions of globular and fibrillar lysozyme were titrated by the successive additions of AuNPs. The mass ratio of AuNPs/lysozyme was varied from 0 to 3 wt% and the variation of the intrinsic tryptophan fluorescence was measured after each addition. In the concentrations range of AuNPs used in the previous fibrillation experiments, only limited variation of the fluorescence signal was observed. The structural and conformational changes were also monitored by TEM, isothermal titration calorimetry (ITC), fluorescence assays of ThT and tryptophan.

The addition of AuNPs to the fibrillar lysozyme led to a decrease of 80 ± 1% in the ThT fluorescence with no shift in *λ*_max_ (ESI, Fig. S3A†). This significant decrease in ThT fluorescence suggests that the amount of β-sheet structures in the fibrillar lysozyme is reduced in the presence of AuNPs. CD measurements supported this observation (ESI, Fig. S4B†). Furthermore, CD and ThT measurement after the addition of AuNPs to globular lysozyme indicated that no conformational changes were observed for similar mass ratio (ESI, Fig. S4A†).

**Fig. 4:**
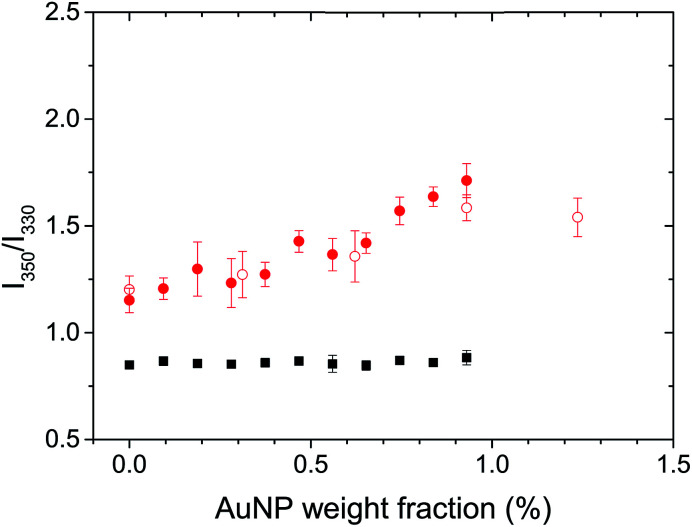
Effect of the addition of gold nanoparticles on the fluorescence of tryptophan in globular lysozyme (black) and in lysozyme fibrils (red). Lysozyme concentration was 2.0 mg mL^−1^ (filled symbols) and 0.6 mg mL^−1^ (open symbols).

The natural fluorescence of proteins is governed by the contribution of tryptophan residues, and the fluorescence of the tryptophan residues is highly sensitive to the presence of different microenvironments within the protein.^38^ Lysozyme presents 6 tryptophan residues.^44^ The *λ*_max_ of the tryptophan fluorescence for globular and fibrillar lysozyme was observed at 334 and 342 nm, respectively (Fig. S3B†). The bathochromic shift and the decrease in fluorescence intensity of tryptophan after the fibrillation of lysozyme were associated with the conformational changes of the protein^38^ leading to the exposure of the tryptophan residues to the environment on the periphery of the fibril. Conformational changes in the protein could be studied by analysing the ratio between the fluorescence of tryptophan at 350 nm and 330 nm (*F*_350_/*F*_330_),^45^ because the local environment of the individual tryptophan residue influence the position of the maximum fluorescence emission, tryptophan located inside of the protein display a *λ*_max_ at lower wavelength (310–330 nm) than tryptophan on the surface interacting with mobile water (340–350 nm).^46^ An increase in *F*_350_/*F*_330_ thus indicated a structural change resulting in a more hydrophilic environment around the tryptophan residues, in average. Fig. 4 shows, a significant difference in the structure of the globular and fibrillar lysozyme in keeping with the formation of β-amyloid fibrils. After fibrillation, the ratio of fluorescence increases from 0.85 ± 0.02 to 1.15 ± 0.06 indicative of the refolding of the protein where the tryptophan residues are in a more polar environment.

The addition of AuNPs to the solution of globular lysozyme, led to no further changes in the ratio of fluorescence, this indicates that the structure of the globular lysozyme was not affected by the addition of AuNPs. However, the addition of AuNPs to the solution of fibrillar lysozyme led to an increase in the ratio of fluorescence, indicative of either further refolding of the lysozyme leading to an increased exposure of the tryptophan residues to a polar environment or to the strong interaction between the tryptophans and AuNPs.

**Fig. 5:**
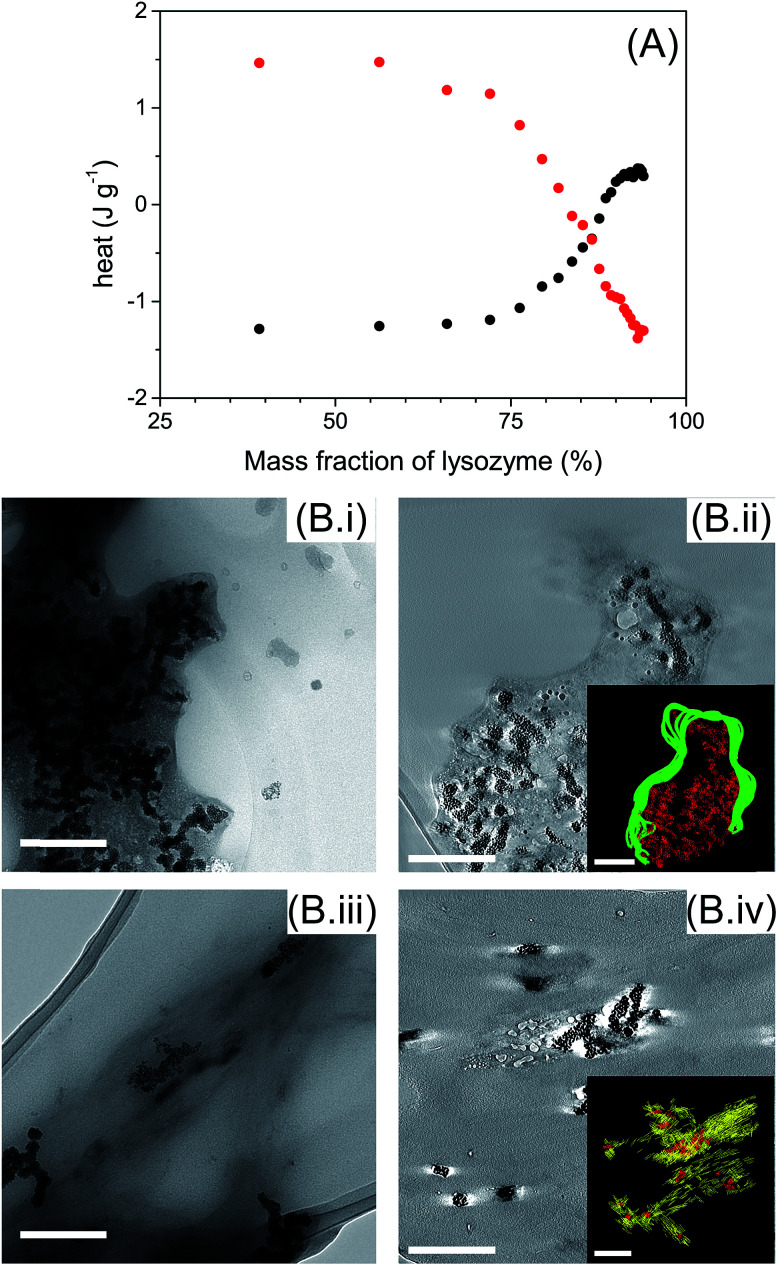
Interaction AuNPs-lysozyme globular and fibrils. (A) ITC data for the adsorption of lysozyme globular (black) and fibrils (red) into AuNPs dispersion. (B) TEM micrographs of ionic liquid embedded samples of AuNPs added in (i and ii) lysozyme globular and (iii and iv) fibrils. (ii) and (iv) are tomogram slices, superimposed by a 3D reconstruction model (yellow = lysozyme fibrils, green = lysozyme globules, red = AuNPs). Scale bar: 200 nm.

ITC measurements were performed to determine the types of interaction involved between AuNPs with globular and fibrillar lysozyme (Fig. 5A). Interestingly, the titration of globular and fibrillar lysozyme with AuNPs suspension exhibited heat variation with opposite behavior. The interaction between the globular lysozyme and the AuNPs was driven by an exothermic process. This suggests that hydrogen bonding and electrostatic interactions between the AuNPs and the protein are the main driving forces in this system,^47,48^ and is coherent with the adsorption of the protein at the nanoparticle interface involving no or limited conformational changes of the protein. On the other hand, the titration of lysozyme fibrils with AuNPs suspension resulted in an endothermic process. This result suggests that the interaction between the AuNPs and the lysozyme fibrils is entropically driven. This nanoparticle–protein interaction is characteristic of the protein desolvation, and the main driving force of this process is the entropy gained by the disorganization of water molecules surrounding the nanoparticles and proteins.^48^ In addition, in endothermic processes, hydrogen bonds interactions are not favoured. This, in conjunction with the distinct tryptophan/AuNPs interaction behaviour (Fig. 4), indicates that the interactions of AuNPs with globular and fibrillar lysozyme are driven by different interaction mechanisms.

TEM images show that AuNPs were surrounded by lysozyme in both globular and fibril structures (Fig. 5B). AuNPs were always located inside a lysozyme-rich environment indicating the affinity of AuNPs with both lysozyme structures. AuNPs were incorporated in globular lysozyme domains (Fig. 5B(i)). No significant morphological changes, other that AuNP inclusion, were observed for the lysozyme fibrils in presence of AuNPs (Fig. 5B(iii)). By TEM tomography, it is notable that AuNPs were located inside lysozyme in both globules (Fig. 5B(ii) inset) and fibrils (Fig. 5B(iv) inset), which confirms the existence of strong interactions between AuNPs and lysozyme in both globular and fibrillar structures. The inclusion of the AuNPs inside the lysozyme fibrils can be associated with the minor disruption of the β-sheet structures (ESI, Fig. S3 and S4†).

## Conclusion

In conclusion, we showed that AuNPs can slow down the lysozyme fibrillation but that once the equilibrium is reached, the content of β-sheet structures formed increased with increasing amount of AuNPs. However, the addition of AuNPs to preformed lysozyme fibrils disrupts the β-sheet structures in the samples as observed by a modification in the fluorescence of the tryptophan residues and circular dichroism. Kinetics studies by TEM images and ThT fluorescence showed that the formation of lysozyme fibrils in presence of AuNPs is impeded resulting in longer lag-time in the protein refolding process. Furthermore, CD measurements confirmed the transition from α-helix to β-sheet conformation during the fibrillation process and that more lysozyme in a β-sheet conformation was observed in presence of AuNPs once the equilibrium was reached. Moreover, the study of interactions between AuNPs and globular and fibrillar lysozyme by ThT and tryptophan fluorescence and confirmed by ITC and TEM images showed different driving forces involved in the interactions between AuNPs and lysozyme. While the addition of AuNPs lead to a modification of the protein structure, the disaggregation of the fibril was not observed. These results illustrate the complex physicochemical interactions involved between AuNPs and amyloid proteins.

## Experimental

### Materials

Tetrachloroauric acid (HAuCl_4_·3H_2_O, 30% in dilute HCl, 99.9%), lysozyme from chicken egg white (lyophilised powder, protein ≥90%), sodium borohydride (NaBH_4_, ≥98%), 3-mercaptopropionic acid (MPA, ≥99%) and thioflavin T (ThT) were purchased from Sigma-Aldrich.

### Gold nanoparticles (AuNPs) synthesis and characterization

The AuNPs synthesis was adapted from a previously described method.^34^ An aliquot of 10 mL of an aqueous solution (0.0875 mmol L^−1^) of HAuCl_4_ was reduced by the addition of 2.4 mL of ice-cold 0.1 mmol L^−1^ NaBH_4_ leading to the formation of AuNPs. The dispersion was stirred at 25 °C for 20 min. Then, 100 μL of 10 mmol L^−1^ MPA was added to the AuNPs dispersion and stirred at 25 °C during 30 min. Subsequently, the dispersion was centrifuged at 14 000 rpm during 3 h to remove the excess of MPA, leaving enough carboxylic groups on the surface of the nanoparticles to provide colloidal stability. The precipitate was washed and redispersed in 1 mL of ultrapure water.

The final concentration of gold in the dispersion is 33.8 mg L^−1^, and was determined by Inductively Coupled Plasma Optical Emission Spectrometry (ICP-OES) (Horiba Jobin Yvon DS 500). The AuNPs were characterised by UV-Vis spectroscopy (Tecan infinite M1000 plate reader), Transmission Electron Microscopy (TEM) (FEI Tecnai F20) and Dynamic Light Scattering (DLS) (PSS Nicomp Submicron Particle Sizer).

### Lysozyme fibrillation

Lysozyme solution (2.0 mg mL^−1^) was prepared in PBS buffer (1.4 mmol L^−1^ KH_2_PO_4_, 8 mmol L^−1^ Na_2_PO_4_, 140 mmol L^−1^ NaCl, 2.7 mmol L^−1^ KCl) at pH 7.3. The concentration of the lysozyme solution was determined spectrophotometrically by its absorbance at 280 nm using an extinction coefficient of *ε* = 38 940 mol^−1^ L^−1^ cm^−1^.^15,39,49^ To produce the amyloid fibrils, the pH of the solution was adjusted to 2.0 by the dropwise addition of HCl, and then agitated at 500 rpm and 60 °C overnight in a ThermoMixer (HLC, MKR23). Different amounts of AuNPs were added to lysozyme solution before the start of the fibrillation, with a final concentration of 0.07, 0.33 and 3.1 mg L^−1^. To follow the amyloid fibrils kinetics, 10 μL of each sample was added to 90 μL of 20 μmol L^−1^ Thioflavin T (ThT) solution at different times during the fibrillation. The ThT was excited at 440 nm and the emission spectra recorded from 450 to 600 nm using both excitation and emission bandwidth of 5 nm. The measurements were done using a plate reader (Tecan infinite M1000 plate reader). All measurements were done in triplicate and the fluorescence intensities were corrected for the PBS buffer blank.

### Tryptophan fluorescence

Aliquots of 5 μL of 33.8 mg L^−1^ AuNPs were added in 90 μL of globular or fibrillar lysozyme and the tryptophan fluorescence spectra were acquired. The tryptophan fluorescence was excited at 295 nm and the emission spectra recorded from 300 to 450 nm using both excitation and emission bandwidth of 5 nm. The fluorescence intensity values were obtained by the integration of spectra in the range 320 to 380 nm. The measurements were done using a plate reader (Tecan infinite M1000 plate reader). All measurements were done in triplicate and the fluorescence intensities were corrected for the PBS buffer blank.

### Circular dichroism (CD) spectroscopy

CD measurements were done with samples of 0.5 mg mL^−1^ protein concentration previously diluted in PBS buffer. Spectra were recorded in the range 200–260 nm using a bandwidth of 1 nm, and a data pitcher of 0.5 nm, with a scan speed of 50 nm min^−1^. All measurements were done in triplicate and the spectra CD were corrected by the PBS buffer blank. The spectra were displayed in molar residue ellipticity (MRE) obtained by the relation:
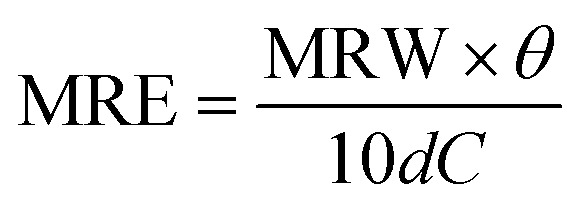
where, *θ* is the ellipticity in degrees, *d* is the cell path length in centimeters and *C* is the concentration in g mL^−1^. MRW is the mean residue weight and is defined as MRW = *M*/(*N* − 1), where *M* is the molecular mass in dalton and *N* is the number of amino acids of the protein. For lysozyme, *M* is 14 300 and *N* is 129. The measurements were done in a JASCO J-815 CD Spectrometer.

### Transmission electron microscopy (TEM) and tomography

The samples were embedded in a matrix of 1-ethyl-3-methylimidazolium tetra-fluoroborate (ionic liquid EMI-BF4) (Sigma-Aldrich). 1 μL of each sample was placed on a copper grid and left to dry. The measurements were performed with a transmission electron microscope FEI Tecnai F20. The images were recorded on a 2k CCD (charge-coupled device) camera (Gatan Ultrascan 1000). More details of the preparation are reported elsewhere.^50^ For tomography, tilt series were recorded from −65° to +65°. The alignments and the weighted back-projection-based reconstructions were computed with eTomo (a program from the IMOD software package^51^).

### Isothermal titration calorimetry (ITC)

ITC measurements were done by the titration of 25 aliquots of 2 μL each one by an automatic injection syringe containing 50 μL of lysozyme globular or fibrils 1.1 mg mL^−1^ into a calorimetry cell with 300 μL of AuNPs 0.0114 g L^−1^. The injections were performed at 250 s interval with stirring at 350 rpm and constant temperature at 25 °C. The heat per injection was subtracted from the heat of dilution of lysozyme titration into water. The measurements were done using a Nano ITC (TA instruments).

## Conflicts of interest

There are no conflicts to declare.

## Supplementary Material

RA-008-C7RA11219J-s001
