# Supplementary material for: Gold nanocolloid–protein interactions and their impact on β-sheet amyloid fibril formation
Source: RSC Adv. 2018 Jan 3;8(2):980–6. doi: 10.1039/c7ra11219j (PMC9077019; doi:10.1039/c7ra11219j)
Supplement: RA-008-C7RA11219J-s001 [file RA-008-C7RA11219J-s001.pdf]

Electronic Supplementary Material (ESI) for RSC Advances.
This journal is © The Royal Society of Chemistry 2017

ELECTRONIC SUPPLEMENTARY INFORMATION

Gold nanocolloids-protein interactions and their impact on β-sheet amyloid fibrils formation

Heloise R. Barros,[a,b] Maria Kokkinopoulou,[a] Izabel C. Riegel-Vidotti,[b] Katharina Landfester,[a] and Héloïse Thérien-Aubin[a]

1. Synthesis and characterization of the AuNPs

AuNPs were synthesized in presence of mercaptopropionic acid, a thiolated molecule providing carboxylic acid to the surface of the nanoparticles. The AuNPs presented typical surface plasmon (SPR) band in the UV-Vis spectrum centered around 550 nm (Fig. S1A). TEM image shows that the obtained AuNPs are small particles with a diameter of 4.4 ± 0.1 nm. The size distribution was supported by DLS measurements showing that the colloidal dispersion has particles with diameters of 3–5 nm and some aggregates of up to 100 nm.

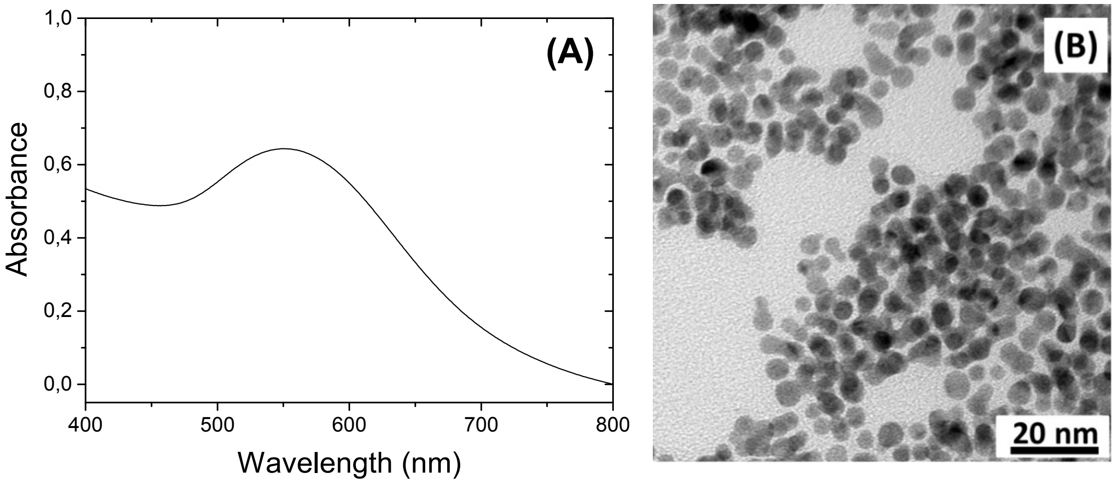

Fig. S1 UV-Vis absorption spectrum of AuNPs (A) and their correspondent TEM image (B).

2. Tomography

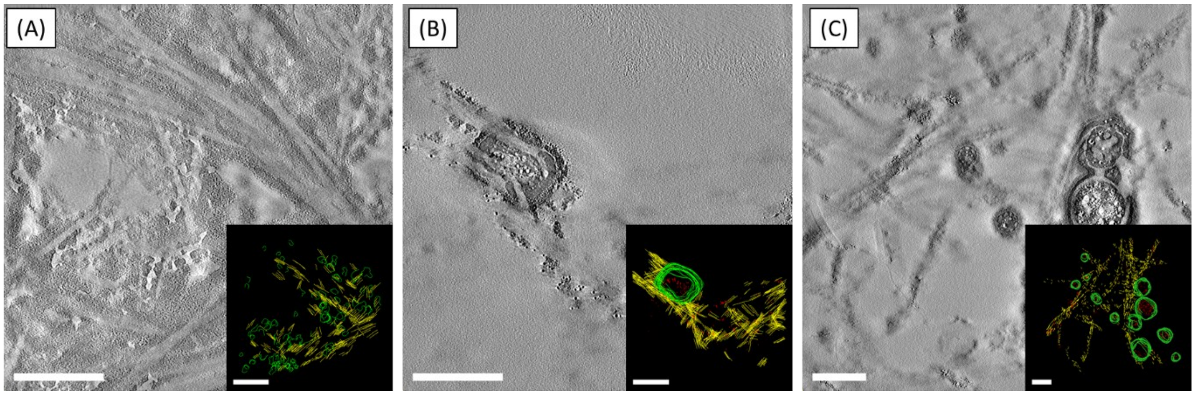

Fig. S2 3D reconstruction of the regions showing the lysozyme fibrillation in the presence of AuNPs. Tomogram slices of ionic liquid embedded samples, prepared during the lysozyme fibrillation at $t_{SA} = 0.5$ with (A) $c_{\mathrm{AuNP}} = 0$ mgL^{-1}, (B) $c_{\mathrm{AuNP}} = 0.33$ mgL^{-1} and (C) $c_{\mathrm{AuNP}} = 3.1$ mgL^{-1}, superimposed by a 3D reconstruction model (yellow = lysozyme fibrils, green = lysozyme globules, red = AuNPs). Scale bar: 200 nm.

3. ThT fluorescence kinetics

Table S1. Kinetics parameters of fibrils formation in presence AuNPs.[a]

Sample	k (min^{-1})	t_{lag} (min)
$c_{\mathrm{AuNP}} = 0$ mgL^{-1}	0.21 ± 0.02	35 ± 1
$c_{\mathrm{AuNP}} = 0.07$ mgL^{-1}	0.13 ± 0.06	80 ± 5
$c_{\mathrm{AuNP}} = 0.33$ mgL^{-1}	0.15 ± 0.02	55 ± 3
$c_{\mathrm{AuNP}} = 3.1$ mgL^{-1}	0.15 ± 0.02	56 ± 2

[a] Values obtained from the fit presented in Figure 2 using Equation 1.

4. Interaction between lysozyme fibrils and AuNPs fluorescence assays

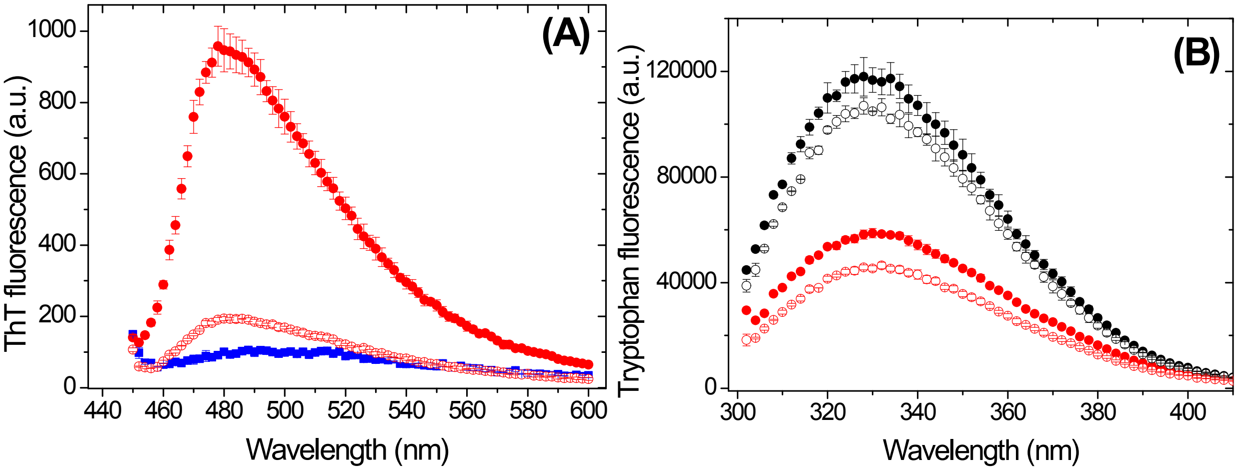

Fig. S3 Fluorescence emission spectra of (A) ThT in water (blue circles), ThT in lysozyme fibrils with $w_{AuNP} = 0$ % (closed red circles) and in lysozyme fibrils with $w_{AuNP} = 0.93$ % (open red circles). $\lambda_{ex} = 440$ nm and $\lambda_{em} = 480$ nm. (B) Tryptophan emission spectra of lysozyme globular (black) and fibrils (red) with $w_{AuNP} = 0$ % (closed circles) and $w_{AuNP} = 0.93$ % (open circles). $\lambda_{ex} = 295$ nm and $\lambda_{em} = 300 - 450$ nm.

5. Circular dichroism of globular and fibrillary lysozyme in presence of AuNPs

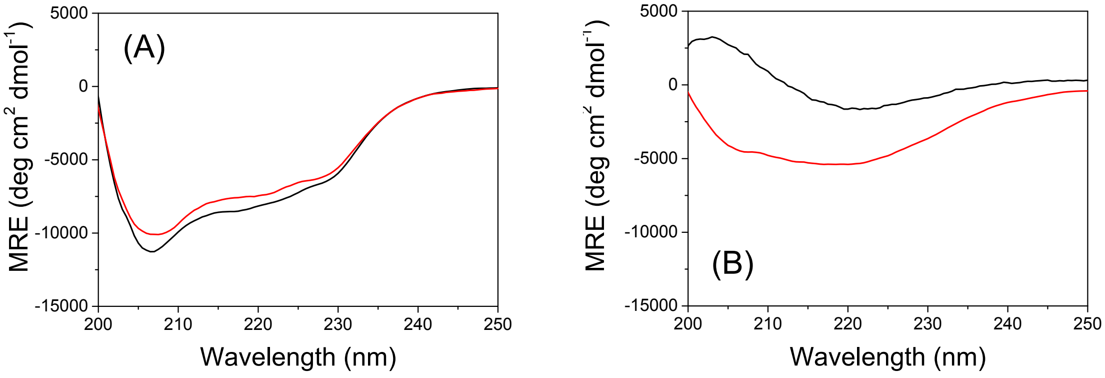

Fig. S4 CD spectra of lysozyme (A) Spectrum of globular lysozyme without AuNPs (black) and after the addition of $w_{AuNP} = 0.93$ % (red). (B) Spectrum of fibrillar lysozyme without AuNPs (black) and after the addition of $w_{AuNP} = 0.93$ % (red).

6. Electrophoresis of lysozyme

Lysozyme was analysed by electrophoresis before and after fibrillation. To performed electrophoresis under reducing condition, an aliquot of the protein sample was mixed with NuPAGE LDS Sample Buffer and NuPAGE Sample Reducing Agent and applied onto a NuPAGE 10 % Bis-Tris Protein Gel (Thermo Fisher Scientific). The gels were loaded with ca. 5 or 10 ug of protein. The electrophoresis was carried out in NuPAGE MES SDS Running Buffer at 100 V for 2 h with SeeBlue Plus2 Pre-Stained Standard (Invitrogen) as a molecular marker. The gel was stained using SimplyBlue SafeStain (Thermo Fisher Scientific).

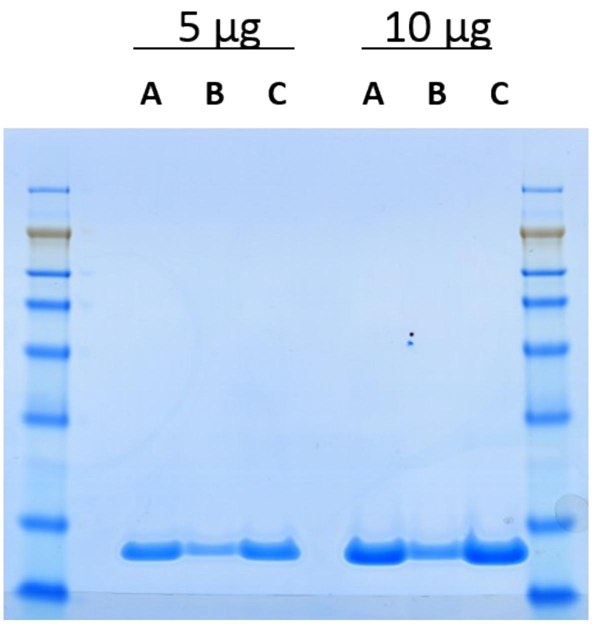

Fig. S5 Polyacrylamide gel electrophoresis (PAGE) of the protein. (A) native protein in PBS buffer at pH7. (B) Globular protein incubated in PBS (pH=7) at 60°C overnight. (C) Fibrils obtained from the incubation of the protein solution in buffer (pH=2) at 60°C overnight.
